# The impact of FSH receptor polymorphism on time-to-pregnancy: a cross-sectional single-centre study

**DOI:** 10.1186/s12884-018-1910-2

**Published:** 2018-06-28

**Authors:** Birute Zilaitiene, Marius Dirzauskas, Rasa Verkauskiene, Rytas Ostrauskas, Joerg Gromoll, Eberhard Nieschlag

**Affiliations:** 1 Institute of Endocrinology, Lithuanian University of Health Sciences, 50009 Kaunas, Lithuania; 2 Center of Reproductive Medicine and Andrology, University of Münster, 48149Münster, Münster, Germany

**Keywords:** Time to pregnancy, FSH receptor haplotypes, FSH receptor polymorphism

## Abstract

**Background:**

Single nucleotide polymorphism of the follicle-stimulating hormone (FSH) receptor (FSHR) is an important marker of ovarian function. However, its role in female fecundity remains debatable. The aim of the study to assess the relationship of FSHR polymorphism of *Serine/Serine*, *Asparagine/Asparagine* and *Asparagine/Serine* variants directly against the time-to-pregnancy (TTP) in women.

**Methods:**

Data were collected from 291 consecutive selected post-partum Caucasians using this criterion: ethnicity, age between 21 and 34-year-old new mothers and, 0–3 days after delivery of newborns in the Klaipeda University Hospital, Lithuania. Questionnaires on factors associated with conception were given to patients, and blood samples were collected for genomic *DNA* extractions as well as for analysis of follicle-stimulating hormone receptor gene polymorphism. Odds ratios (OR) and 95% confidence intervals (CI) for time-to-pregnancy were estimated by multivariate logistic regression. Women with unplanned pregnancies and those who received assisted reproductive technologies were not included in the study.

**Results:**

After adjustment for other possible factors, increased risk for time-to-pregnancy of 12 or more months was associated with: *Serine/Serine* polymorphism variant (OR = 1.38, 95% CI 1.56–2.71, *p* = 0.007), age of 30 or more years (OR = 1.95, 95% CI 1.25–2.71, *p* = 0.015), gynaecological diseases in the past (OR = 2.21, 95% CI 1.12–5.74, *p* = 0.027), prior contraception use (OR = 1.87, 95% CI 1.14–3.64, *p* = 0.016), and fertility problems in the past (OR = 1.57, 95% CI 1.16–4.76, *p* = 0.019).

**Conclusion:**

The results suggest a possible relationship of FSH receptor gene *Serine/Serine* variant for the lower possibility of conception during the first 12 months of planned conception.

## Background

Fecundity is the wonderful biological ability to produce abundant healthy offspring and is affected by genetic and environmental factors [1]. If pregnancy is planned, fertility may be expressed as time-to-pregnancy (TTP) [2]. TTP is defined as the number of contraceptive-free cycles needed to conceive [3]. A TTP greater than 12 months allocates the infertility status [4, 5]. Usually trying to conceive in the first year succeeds for ~ 85% of cases [6].

Impaired fertility is inherited and may be due to inactivating mutations in the gonadotropin and gonadotropin receptor genes [7, 8]. Recent genetic studies have revealed that the pathogenesis of subfertility or infertility can be due to mutations in the follicle-stimulating hormone receptor (FSHR) gene [9]. While mutations affecting FSHR are sporadic; polymorphism of the FSHR gene seems to be a common phenomenon [9]. FSHR inactivating mutations may cause primary or secondary amenorrhea, infertility, and premature ovarian failure [10]; whereas activating mutations can predispose to ovarian hyperstimulation syndrome, as a consequence of exogenous FSH administration, or to a spontaneous onset [10–12].

In-vitro studies have shown that the A allele at the 29th position in the 5′ untranslated region of the FSHR gene is associated with impaired transcriptional activity [13]. The polymorphism at position 29 in the promoter of the FSHR gene may contribute to the reduced receptor expression [14]. The FSHR shows nucleotide polymorphisms in the promoter and in exon 10 [15]. The single nucleotide polymorphisms in exon 10 results in four discrete allelic variants characterized by the amino acid combinations: *threonine (Thr)307*-*asparagine (Asn)680, alanine (Ala)307*- *Serine* (Ser)680, *Ala307-Asn680* and *Thr307-Ser680* [15]. The first two allelic variants are very frequent in the Caucasian population [15]. At position 680, three FSH receptor variants are possible: *Asn/Asn, Asn/Ser,* and *Ser/Ser;* however, *Ser/Ser-680* predominates in the studied infertile population [16].

The studies on FSHR polymorphism, performed on patients undergoing in-vitro fertilisation procedures show that women homozygous for the *Ser680* variant have higher follicular FSH levels and longer follicular phase length, which suggest a lower sensitivity to FSH. Thus the homozygous *Ala307-Ser680* variant is associated with a higher amount of FSH required for ovarian stimulation in women undergoing assisted reproduction [15]. This suggests that the FSHR genotype can influence the ovarian response to FSH stimulation [17, 18]. However, there are studies where this association was not confirmed [19, 20].

FSH is responsible for follicular maturation and for the length and stability of the menstrual cycle [21]. A longer cycle may be associated with more difficulties in conception; women who have the FSHR gene *Asn (Asparagine)* exchanged for *Ser (Serine)* at codon 680 have statistically proven longer menstrual cycles [22].

Despite the numerous publications on the FSHR polymorphism impact on women’s reproductive function; an FSHR polymorphic relationship to TTP has not yet been studied. Here we aimed to assess the relationship of the FSHR polymorphism Serine/Serine, Asparagine/Asparagine and Asparagine/Serine variants on TTP in a sample of Lithuanian women.

## Methods

### Subjects

Klaipeda is the third largest city in Lithuania and has one obstetric department that performs approximately 3500 deliveries per year from all parts of the west region of Lithuania. Between March 2008 and May 2008, 291 consecutive selected 21–34-year-old (mediana [25–75%] – 27.0 [24.0–31.0] years) women who conceived naturally and delivered babies at the Klaipeda University Hospital were invited to participate in the study 0–3 days after delivery. This time interval was chosen because it allowed for the accurate recall of the time period preceding conception, and it was a time point at which the delivery outcome was already known. All selected women had planned pregnancies that were achieved without using assisted reproductive technologies. In 49.17% of cases women were nulliparous, the rest of them - multiparous. Prior to pregnancy planning, 67 (22.26%) women used hormonal contraception. Women were asked to complete a standardised questionnaire. It included questions concerning age, height, weight before pregnancy, the menstrual cycle, socioeconomic factors, lifestyle, sexual behaviour, and some other factors. The regular menstrual cycle was defined as 28 ± 7 days and this definition was explained to study participants. Women with irregular menstrual cycles were asked to report if the majority of their cycles are < 21 days or > 35 days. Only one participant reported irregular cycles < 21 days, so, she was excluded from the final analysis. The questions regarding gynaecological diseases were asked separately for non-infectious and infectious diseases. Women with unplanned pregnancies and those who received assisted reproductive technologies were not included in the study. However, women who get another infertility treatment, e.g. ovulation induction, treatment of infections - were enrolled into the study.

### DNA sampling

A venous blood sample was drawn for DNA extraction from all 291participants.

*DNA* extraction was performed in a certificated “SORPO” laboratory of Thermo Fisher Scientific Inc. in Vilnius, Lithuania. *DNA* samples froze at-20 °C; were sent to the University of Munster in Germany. There are two known polymorphisms of clinical relevance in the hormone (FSH) receptor exon 10: *Ala* or *Thr* at position 307 (dbSNP numbers 6165), and *Asn* or *Ser* at position 680 (dbSNP numbers 6166). These give rise to two discrete allelic variants: *Thr*^*307*^*/Asn*^*680*^ and *Ala*^*307*^*/Ser*^*680*^. The allelic variants at codon 307 and 680 are almost invariably associated, therefore codon 680 was assessed, and all women were classified as homozygous (*Ser/Ser* or *Asn/Asn*) or heterozygous (*Asn/Ser*).

Genomic *DNA* was extracted from peripheral blood using a FlexiGene *DNA* extraction kit (QIAGEN, Hilden, Germany) according to the manufacturer’s instruction. All samples were screened for the single nucleotide polymorphism (SNP) at position 2039 (codon 680) of exon 10 by the TaqMan allelic discrimination assay while using the ABI Prism 7000 sequence detection system (Applied Biosystems, Darmstadt, Germany). The probes (SNP indicated in *bold lower case letters*) were 5′-*AGAGTCACCA****g****TGGTT*-3′ (6-carboxyfluorescein fluorescence) and 5′-*AGTCACCA****a****TGGTTC*-3′ (VIC fluorescence). The primers were 5′-*AAGGAATGGCCACTGCTCTTC*-3′ (forward) and 5′-*GGGCTAAATGACTTAGAGGGACAA*-3′ (reverse). Each polymerase chain reaction (PCR) (25 μl) contained: 2 μl DEPC-treated water, 12.5 μl Universal master mix, 0.25 μl of each probe, and 4.5 μl of each primer (5 pmol). Using the TaqManassay, PCR was performed in two steps: absolute quantification and allelic discrimination. For absolute quantification, the cycles were as follows: stage 1: Probe binding at 50 °C for 2 mins (1 cycle); stage 2: denaturation at 95 °C for 10 mins (1 cycle), followed by 35 cycles at 95 °C for 15 s; stage 3:60 °C for 1 min. Whereas the allelic discrimination assay took 1 min at 60 °C.

### Statistical analysis

Analyses were performed using SPSS 17.0 software. Women who conceived after ≥12 months of trying were classified as the risk group. The normality of distribution was tested using the Kolmogorov-Smirnov test. Student’s (t) criterion was used for comparison of means for normal distributions, and the Mann-Whitney (U) test was used in skewed distributions. In order to determine the difference between more than two groups; parametric and nonparametric dispersive analysis with ANOVA and Kruskal-Wallis test was performed. Bonferroni test was performed by comparing multiple pairs. For evaluating dependence between qualitative features χ^2^ criterion was used. Results were presented as mean (M) ± standard deviation (SD) or n (%). Biological, social, demographic, economic, sexual behaviour, genetic, living, working and environmental confounders variables were retaining in models. Univariate analysis of the OR for each variable was taken initially. Multivariate logistic regression step-wise enter method model was used to estimate the most important relationship factors. Odds ratios (OR) and 95% confidence intervals (CI) for time-to-pregnancy was calculated. The limit of significance was defined as a two-sided *p*-value of< 0.05.

The study was approved by the Lithuanian Bioethics Committee (21/12/2006 No. 59/2). The aim of the survey protocol was carefully explained to each subject of study entry, and a written informed consent was obtained.

## Results

### FSHR genetic variants

The mean TTP in the study group of 291 woman was 5.3 ± 10.9 (mediana [25–75%]: 1.0 [1.0–5.0]) months. The main demographic, social, lifestyle and other characteristics of the participants are shown in Table 1.

**Table 1:** Main characteristics of study participants

Criteria	FSHR genetic variant					
	*Asn/Asn*		*Asn/Ser*		*Ser/Ser*	
	n	% or Median (25–75% CI)	n	% or Median (25–75% CI)	n	% or Median (25–75% CI)
Participants	101	34.7	148	50.9	42	14.4
Mean age (years)		27.4 ± 5.9		27.2 ± 5.5		27.9 ± 5.0
Median body mass index (kg/m^2^)		21.4 (19.8–24.1)		22.3 (20.0–24.1)		21.5 (20.1–23.1)
Median TTP (month)		1.0 (1.0–4.0)*		1.0 (1.0–3.8)*		7.0 (1.0–15.3)
TTP < 12 month	92	91.1*	133	89.9*	27	64.3
TTP ≥ 12 month	9	8.9	15	10.1	15	35.7
Nuliparous	48	47.5	75	50.7	25	59.5
Multiparous	53	52.5	73	49.3	17	40.5
Living in the city	76	75.3	115	77.7	35	83.3
Living in the country	25	24.7	33	22.3	7	16.7
Education lower than college	37	36.6	63	42.6	14	33.3
College education and higher	64	63.4	85	57.4	28	66.7
Salary < 560 Euro/month	34	33.7	36	24.3	11	26.2
Salary ≥560 Euros/month	67	66.3	112	75.7	31	73.8
Smoking	28	27.7	28	18.9	6	14.3
Alcohol consumers	51	50.5	74	50.0	20	47.6
Coffee consumption	27	26.7*	37	25.0*	32	76.2
Folic acid use	24	23.8	36	24.3	11	26.2
Use of other food additives	32	31.7	50	33.8	14	33.3
Physical activity/sports	28	27.7	48	32.4	16	38.1
Prior hormonal contraception use	26	25.7	31	20.9	10	23.8
Regular menstrual cycle	68	67.3*	113	76.4*	17	40.5
Irregular menstrual cycle	33	32.7*	35	23.6*	25	59.5
Sexual intercourse one time/week	18	17.8	27	18.2	7	16.7
Sexual intercourse 2 times and more/week	83	82.2	121	81.8	35	83.3
Past fertility problems	8	7.9	6	4.0	3	7.1
Gynaecological diseases in the past	3	3.0	4	2.7	1	2.4
Working status	93	92.1	141	95.3	39	92.9
Stress	60	59.4	55	37.2	21	50.0
Use of pesticides	2	1.9	1	0.7	1	2.4

n – number of study participants; ^*^p < 0.05 compared with the *Ser/Ser* group. For quantitative variables p value by non-parametric ANOVA (Kruskal Wallis), for qualitative variables *p* value by χ^2^ test

During FSH receptor genotype analysis three groups of *Asn*^680^ and *Ser*^680^ variation were detected: 101 (34.7%) of cases were found to be homozygous for *Asn*^680^ (*Asn/Asn* -group), 148 (50.9%) heterozygous for *Asn*^680^ and *Ser*^680^ (*Asn/Ser* -group), and 42 (14.4%) homozygous for *Ser*^*680*^ (*Ser/Ser* -group). Median TTP in the *Asn/Asn* participant group was 1.0 [95% CI 1.0–4.0] months, in the *Asn/Ser* group: 1.0 [1.0–3.75] months, and in the *Ser/Ser* group: 7.0 [1.0–15.25] months. Furthermore, these differences of were significant (*p* < 0.03).

**Fig. 1:**
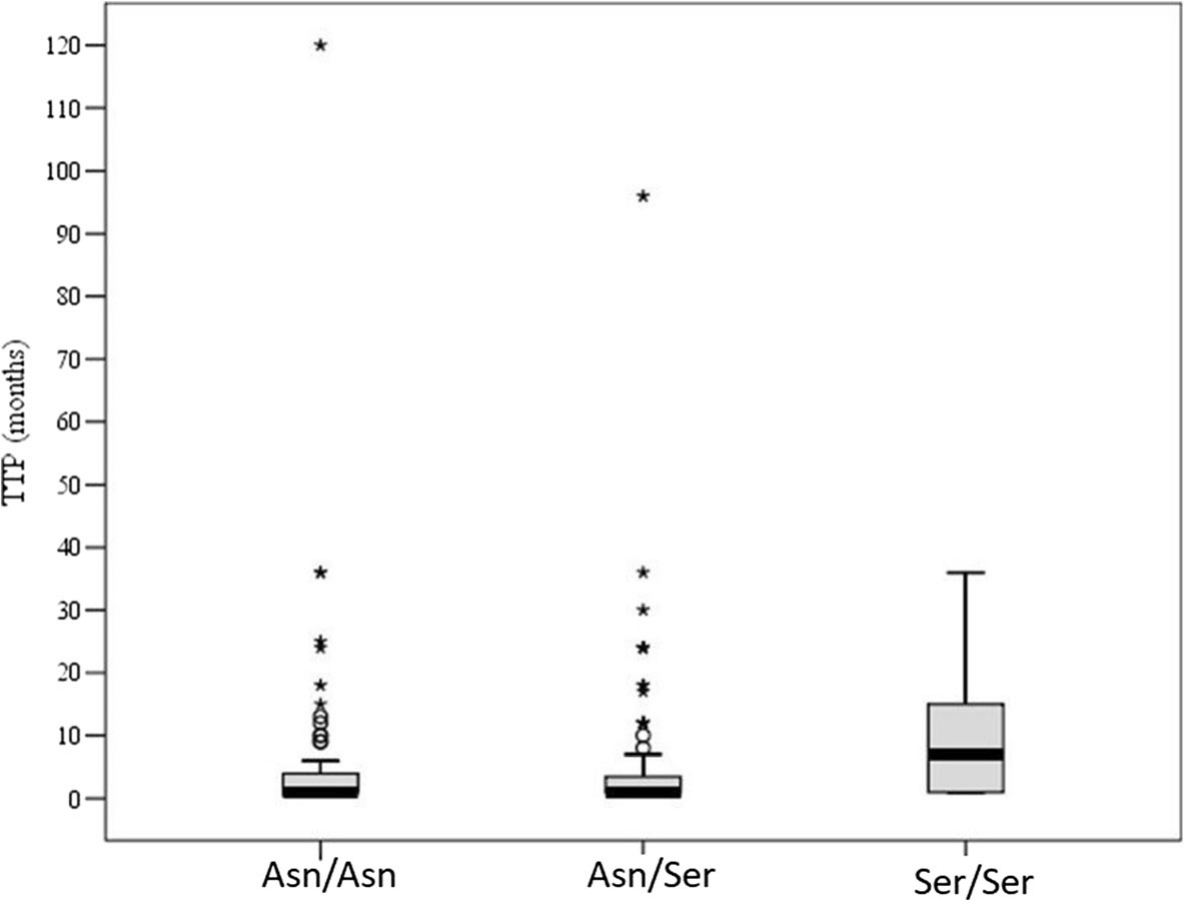
Distribution of the FSHR polymorphism in TTP groups

Women having the *Ser/Ser* polymorphism variant had significantly longer TTP compared to those bearing variants *Asn/Asn* and *Asn/Ser* (*p* = 0.01 and p = 0.01 respectively) (Fig. 1). When comparing *Asn/Asn* and *Asn/Ser* groups, no significant differences in TTP were found.

In the comparison between the *Ser/Ser* and the *Asn/Asn* FSHR genetic variants, the risk for a TTP of 12 months or longer is higher for those women with the variant *Ser/Ser* (Table 2). The highest sensitivity and positive prognostic value, along with the lowest false positive rate α, occur when comparing the Ser/Ser genetic variant to *Asn/Asn*. Thus this combination has the highest effect size for estimating TTP (Table 3).

**Table 2:** Estimated odds ratios for TTP ≥12 months for FSHR genetic variants

	OR	95% CI	*P*-value
*Ser/Ser* vs. *Asn/Asn*	5.68	4.83–6.70	< 0.0001
*Ser/Ser* vs. *Asn/Ser*	4.93	4.28–5.68	< 0.0001
*Ser/Ser* vs. *Asn/Asn* + *Asn/Ser*	5.21	4.65–5.85	< 0.0001

OR – odds ratio*,* p – significance level

**Table 3:** Prognostic value of FSHR polymorphism variants for TTP < 12 vs. ≥12 months

	Sensitivity (%)	Specificity (%)	Prognostic value		False rate	
			Positive (%)	Negative (%)	Positive (α)	Negative (β)
*Ser/Ser* vs. *Asn/Asn*	62.50	77.31	65.71	8.91	22.69	37.50
*Ser/Ser* vs. *Asn/Ser*	50.00	83.13	49.15	10.14	16.88	50.00
*Ser/Ser* vs. *Asn/Asn* + *Asn/Ser*	38.46	89.29	54.78	9.64	10.71	61.54

Women with the *Ser/Ser* polymorphism had irregular menstrual cycles (> 35 days) more frequently when compared to other genetic variants (χ^2^ = 20.2; df = 2; *p* < 0.001) (Table 1). The highest odds ratio is found when comparing *Ser/Ser* and *Asn/Ser* FSHR genetic variants, and the risk for having irregular menstrual cycle is 4 times higher in women with the *Ser/Ser* variant.

### Risk factors for a TTP of 12 or more months for the women in the study group

Logistic regression methods were used to explore potential risk factors for longer TTP. Proportional differences were analysed to compare data of women who conceived up till 12 months with data of women who conceived at 12 or more months. Meanwhile, other factors possibly having an influence on TTP were also checked in this study. Most of the study participants were living in the city, in their own residences, as couples had higher education, higher monthly salary, worked, didn’t smoke, and drank coffee (Table 1). Only a few of cases were obese (5.50%), had previous gynaecological diseases (15.46%), or fertility problems (5.84%). Respondents with alcohol consumption reported stress during pregnancy planning and pregnancy quantity; both divided up equally. The use of folic acid or other food supplements was surprisingly low (24.40 and 33.33% respectively). TTP of ≥12 months was reported significantly more often by women whose age was 30 years or more (*p* = 0.048), who had irregular menstrual cycles (*p* < 0.001), previous fertility problems and/or gynaecological diseases (p < 0.001 both), used any contraception prior to pregnancy planning (*p* = 0.004), drank coffee (p = 0.048), consumed other food supplements (p = 0.004), lived within < 10 km from factories (p = 0.04), had low physical activity (*p* = 0.044), and the SER/SER polymorphism variant (*p* < 0.001). Unadjusted univariate OR and 95% CI for conceiving after 12 or more months with the presence of previously mentioned factors are presented in Table 4.

**Table 4:** Univariate odds ratios for TTP ≥12 months in the group of women analysed for FSHR polymorphism

Variables^a^	OR	95% CI	p
Past fertility problems	6.97	6.22–7.82	< 0.0001
Prior contraception use	6.43	5.74–7.22	0.0043
Irregular menstrual cycle	4.24	3.79–4.77	< 0.0001
Gynaecological diseases in the past	3.44	3.07–3.86	0.0009
Living 10 or less km from factories	2.06	1.84–2.32	0.0399
Age 30 years and older	1.31	1.17–1.47	0.0477
*Ser*/*Ser* polymorphism variant	5.20	2.45–11.05	0.0004

p – significance level, OR – odds ratio (unadjusted), CI – confidence intervals, ^a^ - only significative variables were presented in the table

Coffee consumption and low physical activity correlated significantly with fertility problems in the past (*r* = 0.2; *p* = 0.001 and *r* = 0.23; *p* = 0.013 respectively); meanwhile, irregular menstrual cycles correlated with FSHR gene Ser/Ser variant (*r* = 0.17; *p* = 0.008); a ≥ 560 EURO monthly salary and the use of other food additives – with older age (r = 0.17; p = 0.008 and *r* = 0.27; *p* = 0.03 respectively); as well as living < 10 km from factories, which correlated with gynaecological diseases (*r* = 0.18; *p* = 0.009); therefore, these factors were excluded from further analysis. The use of contraception prior to pregnancy planning showed no correlation to other factors from the univariate regression model. The evaluation of significant correlations was done using the Forward Stepwise likelihood ratio method and was referenced to the database. This algorithm converged through 3 steps; selecting: older age (≥30 years), the use of any contraception prior to pregnancy planning, previous fertility problems, gynaecological diseases, and the *Ser/Ser* polymorphism. These were the most significant factors that correctly predicted TTP of 12 or more months (positively classified prognosis was 91.1%). The combination of these factors formed the multivariate logistic regression model (Table 5). All independent variables were included in the analysis (older age, irregular menstrual cycle, past fertility problems, gynaecological diseases, use of contraception prior to conception, living < 10 km from factories, and having *Ser*/*Ser* polymorphism variant); however, the Forward Stepwise Likelihood ratio method selected the 5 most significant ones (stated above). Accordingly, older age (≥30 years), use of any contraception prior to conception, and having gynaecological diseases increased the OR of conceiving after 12 or more months almost by double; having fertility problems in the past: 1.5 times, and if *Se*r/*Ser* polymorphism is present: 1.7 times.

**Table 5:** A multivariate stepwise Enter model describing significant factors for TTP ≥12 months in the group of women analysed for the FSHR polymorphism

Variables	OR	95% CI	*P*-value
Past fertility problems	1.568	1.16–4.76	0.019
Prior contraception use	1.871	1.14–3.64	0.016
Gynaecological diseases in the past	2.212	1.12–5.74	0.027
Age 30 years and older	1.952	1.25–2.71	0.015
Ser/Ser polymorphism variant	1.678	1.56–2.71	0.007

Constant = 3.741

## Discussion

The FSHR polymorphism’s impact on women’s reproductive function has been demonstrated in several studies [9, 11, 16, 17, 23]; particularly in some diseases, such as the polycystic ovary syndrome and amenorrhea [24–26]. Some investigations provide contradictory data on the relationship between single nucleotide polymorphisms, and their link to polycystic ovary syndrome and amenorrhea [27–29]. The main reported findings on changes of hormonal dynamics in women with homozygote mutated *Ser*^*680*^ throughout the menstrual cycle were with lower serum levels of estradiol, progesterone and inhibin A [22]. However, these women had significantly higher FSH levels, and longer menstrual cycles [12, 14, 18]. Patients with the *Ser*^*680*^*/Ser*^*680*^ genotype are more resistant to FSH action and thus require a stronger stimulus for the same biological response [22]. This finding is important in infertility treatment; patients with the homozygous FSHR *Ser*^*680*^*/Ser*^*680*^ polymorphism have double the chance of having a resistance to clomiphene citrate [30]. They require higher FSH dosages in order to show the same estradiol response during controlled ovarian stimulation [24]. Furthermore, it was also demonstrated that the frequency of *Ser*^*680*^*/Ser*^*680*^ variation in the control population is lower than if compared to the infertile women’s group [23]. Thus it may be hypothesised that the *Ser*^*680*^*/Ser*^*680*^ genotype could be directly related to a women’s fertility. To our knowledge, the FSHR polymorphism was never investigated in direct relation to TTP in a fertile population. In this study, women were considered to be fertile if they achieved pregnancy without using assisted reproductive technology methods. A large number of factors possibly affecting TTP were investigated along with the FSHR polymorphism in order to detect independent factors predicting longer TTP and to establish the role of FSHR polymorphism between other determinants of women’s fertility.

Our data confirmed that higher age, previous gynaecological diseases, and/or fertility problems pose as risk factors for longer TTP. An association for the risk of longer TTP due to hormonal contraception use prior to conception is more questionable. However, an older age of women using contraception could be the reason for this finding.

The relationship of the FSHR polymorphism to the length of the menstrual cycle was demonstrated in our study, as well as in previous publications [22]. Differences in menstrual cycle length between the *Ser*^*680*^*/Ser*^*680*^ and the *Asn*^*680*^*/Asn*^*680*^ groups result in 12.5 vs. 13.5 menstrual cycles per year, respectively [22]. Assuming no difference in age at the time of menopause; women with the *Ser*^*680*^*/Ser*^*680*^ genotype would experience 30–40 cycles fewer, than women with an *Asn*^*680*^*/Asn*^*680*^ genotype during their reproductive life [22]. Some authors conclude that women with the *Ser*^*680*^*/Ser*^*680*^ genotype have a lower chance to achieve pregnancy during the same time period if compared to the other variants [23, 30]. Therefore, menstrual periods are stressful events that have certain disadvantages; such as blood loss, menstrual discomfort, and the effects of hormone fluctuations on mood, breast and other oestrogen-dependent organs. This gives rise to some speculation that fewer menstrual cycles during the reproductive lifespan might represent an evolutionary advantage and might influence fertility positively [22]. Our data provide direct evidence that women with the *Ser*^*680*^*/Ser*^*680*^ genetic variant had a lower chance of conception than females with *Asn*^*680*^*/Asn*^*680*^ and *Asn*^*680*^*/Ser*^*680*^ genetic variants.

We have demonstrated that the FSH receptor gene *Serine/Serine* variant polymorphism is associated with a fivefold lower likelihood to become pregnant during the first 12 months of attempts to conceive.

The other independent factors predicting a TTP of 12 or more months in the study group were older age, gynaecological diseases, fertility problems in the past, and the use of contraception prior to conception.

Some limitations of our study, especially related to the retrospective design, should be discussed. A retrospective design of the study was used in order to achieve a higher participation rate. Only one polymorphism in this region was evaluated, and furthermore, no replication in an independent cohort was attempted. However, it was previously demonstrated that immediately after delivery women can recall the period before conception very well, so data reported here can be treated as reliable [2]. Because of the selected study design, it was not possible to include women who had miscarriages, ectopic pregnancies, or an induced abortion; as well as, to collect information on other important factors that may affect TTP, such as basal FSH levels and semen quality. Moreover, the study was conducted in only one region of the country, which represents one quarter of the entire Lithuanian female population.

### Strengths and limitations of this study

- The FSH receptor gene polymorphism may affect human reproduction by causing menstrual cycle disorders.
- The present study demonstrates the effect of FSH receptor gene polymorphism on time to pregnancy* that has not been investigated till now.
- The relationship of FSHr *Serine*^*680*^*/Serine*^*680*^ variant polymorphism to lower fecundity can have clinical relevance; e.g. more conservative infertility management can be suggested for women with unexplained infertility having this genetic variant.
- Further studies including prospective studies on the impact of genetic factors on women’s fertility are needed.

## Conclusions

Further studies including prospective studies on the impact of genetic factors on women’s fertility are needed. Comprehensively study the effects of FSHR polymorphisms on various reproductive traits, the most studied rs6166 SNP should be evaluated together with the rs1394205 in the 5’UTR and with the SNPs in the FSHB locus [31]. However, it is already clear that the relationship of FSHR *Ser*^*680*^*/Ser*^*680*^ variant polymorphism to lower fecundity can have clinical relevance; e.g. more conservative infertility management can be suggested for women with unexplained infertility whose have this genetic variation.

## Abbreviations

5′ UTR: 5′ untranslated region
*Ala*: *Alanine*
*Asn*: *Asparagine*
CI: Confidence interval
FSH: Follicle-stimulating hormone
FSHB: Follicle-stimulating hormone beta subunit
FSHR: Follicle-stimulating hormone receptor
OR: Odds ratios
*Ser*: *Serine*
SNP: Single-nucleotide polymorphism
*Thr*: *Threonine*
TTP: Time-to-pregnancy
